# ‘Immune reset plus’: the case for combining immunotherapies to maintain self-tolerance in autoimmune diseases

**DOI:** 10.3389/fimmu.2025.1634090

**Published:** 2025-07-23

**Authors:** Fabrina Gaspal, Parth Narendran, Sky T. H. Ng, Michael J. Price, David C. Wraith

**Affiliations:** Department of Immunology and Immunotherapy, College of Medicine and Health, University of Birmingham, Birmingham, United Kingdom

**Keywords:** immune reset, immunological tolerance, antigen-specific immunotherapy, B cell, dendritic cell

## Immune reset: introduction

The phrase ‘immune reset’ for treatment of autoimmune diseases implies the restoration of a stable, self-tolerant immune system (1). In practical terms, immune reset currently involves the depletion of a subset of autoreactive lymphocytes in the hope of restoring homeostatic immune function. We know that the immune system in people with autoimmune diseases can reset itself. This is seen, for example, in some women with rheumatoid arthritis (RA) (2) and multiple sclerosis (MS) (3) who achieve relief of symptoms during pregnancy. Sadly, however, this generally does not persist and can be followed by the onset of serious flares in the post-partum period (4). It is also seen in paraneoplastic autoimmune diseases, where treatment of the tumour can result in remission of the associated autoimmunity. For example, Lambert Eaton myasthenic syndrome (LEMS) is associated with small cell lung cancer in approximately 50% of cases (5). Indeed, symptoms of LEMS in affected patients usually occur before discovery of the cancer. It is thought that the expression of voltage gated calcium channels by small cell lung cancer cells provokes the anti-channel antibody response leading to symptoms of LEMS. Hence, tumour treatment improves the symptoms of autoimmune disease by reducing the antigenic stimulus arising from the tumour.

Immune reset can be achieved by drastic disruption of the immune system. For example, myeloablative or non-myeloablative conditioning of patients followed by autologous human stem cell transplantation (aHSCT) can lead to sustained improvement in autoimmune diseases such as multiple sclerosis (6). However, this does not produce remission in all patients and remains associated with non-relapse mortality at a rate of ~1/30 (7). As a result, clinical use of aHSCT for treatment of autoimmune diseases is only available in specialised clinics and does not have regulatory approval in most countries. Alemtuzumab, anti-CD52, antibody treatment leads to ablation of most white blood cells with sustained depletion of CD4^+^ cells for many months (8). Treatment of patients with relapsing multiple sclerosis with a course of alemtuzumab has a dramatic impact on disease progression (9). However, treatment is associated with development of unrelated autoimmune conditions including Graves’ disease and immune thrombocytopenic purpura in treated individuals as the immune system recovers from T cell depletion (10). It seems likely that development of such unrelated autoimmune diseases is due to the impact of the depleting antibody on regulatory T cell populations (11). Results from studies with alemtuzumab warn us that non-discriminate depletion of T cells should be avoided.

## Immune reset: current approaches

Most current immune reset approaches involve depletion of B cells. This seems sensible in antibody-mediated autoimmune conditions such as myasthenia gravis, Graves’ and SLE; however, recent studies have shown that B cell depletion can have a dramatic impact on cell-mediated conditions such as MS (12). It is still not clear why antibodies targeting CD20 should have such an impact on MS. In theory, this could be due to a) depletion of antigen presenting cells (APCs), given the ability of CD20^+^ B cells to present antigens (13, 14) and b) depletion of EBV infected B cells, based on recent evidence that EBV infection has a role in initiation and/or propagation of MS-related immune pathology (15). Anti-CD20 treatment has been approved for RA, pemphigus vulgaris and ANCA-positive vasculitis (16). While anti-CD20 was not effective in SLE and lupus nephritis, anti-CD19 CAR-T cell treatment has shown efficacy in rituximab-resistant patients (17). CAR-T cells have the added advantage of targeting B cells in lymphoid tissues (18). These results emphasise the need to understand the role of distinct B cell subsets in different diseases. More recently, bispecific antibodies targeting CD3^+^ T cells to CD19^+^ B cells have been used to treat blood cancers and have been tested in autoimmune diseases (19). These, along with T cell engaging agents targeting T cells to alternative B cell surface antigens, such as B-cell maturation antigen (BCMA), are being developed (20).

**Figure 1:**
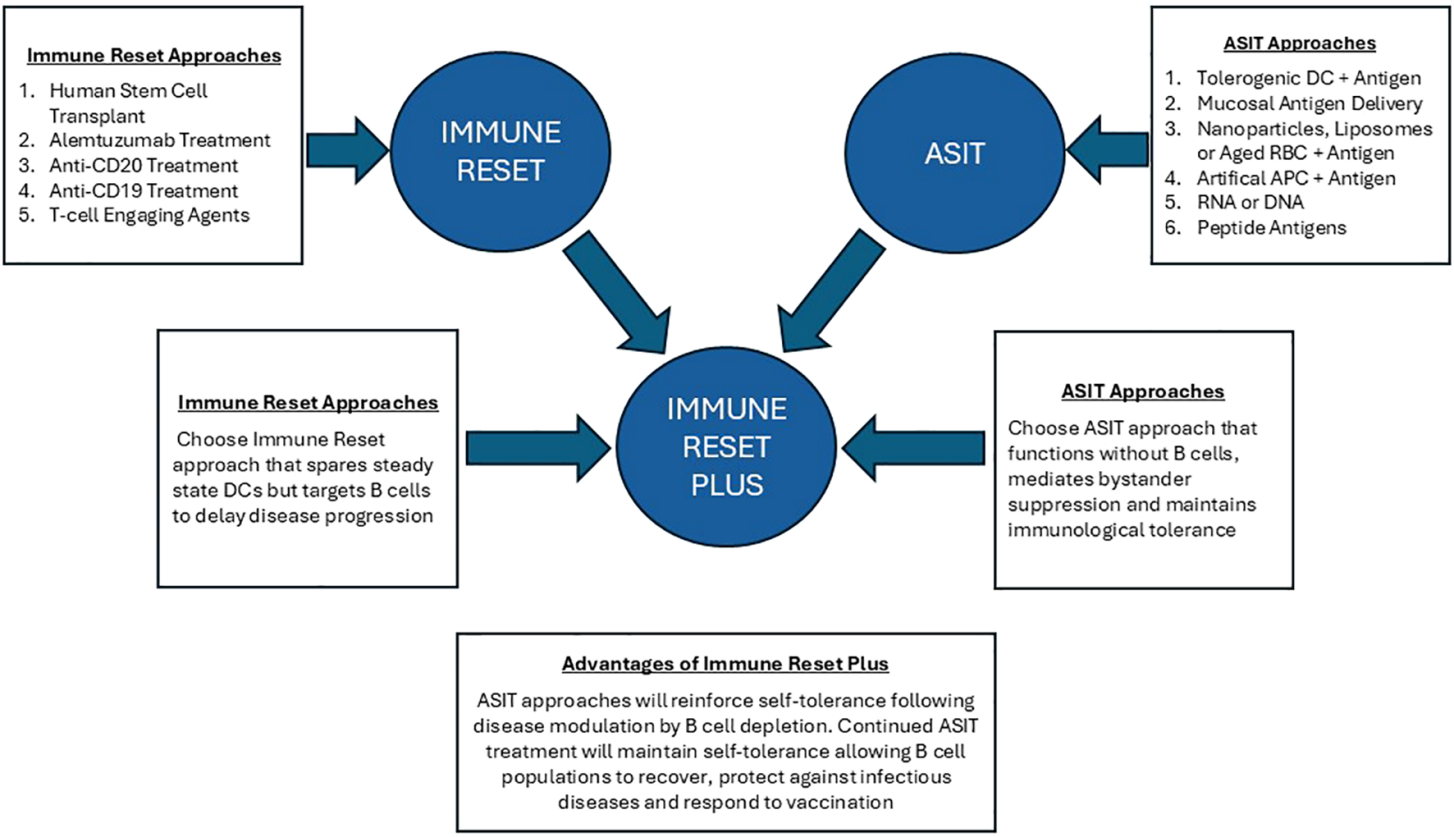
Immune reset plus: this figure illustrates the advantage of combining Immune Reset by B cell depletion with antigen-specific immunotherapy. ASIT with antigens delivered to tolerogenic APC can ‘switch off’ autoreactive T cells while promoting differentiation of antigen-specific regulatory T cell populations. Most importantly, ASIT approaches have been shown to function in B cell depleted animal models. Tolerance induction by ASIT depends on antigen presentation by steady state dendritic cells in lymphoid organs or by APC in the tolerogenic environment of the liver. Immune reset plus ASIT will enable disease to be controlled without continued B cell depletion. This will allow the treated individual to control infections and respond to vaccine.

Does B cell depletion lead to immune reset? The aim of immune reset is to provide restoration of a stable, self-tolerant immune system. However, a single cycle of B cell depletion rarely provides sustained clinical control: anti-B cell approaches generally require continuous treatment for effective disease control (21). It should be noted that long-term B cell lymphopenia increases the risk of microbial infections (22) and hampers effective vaccination, as evidenced in the recent COVID-19 pandemic (23). Alternative approaches are required and here we propose the combined use of B cell depletion plus antigen-specific immunotherapy for stable control of autoimmune diseases (**Figure 1**).

## Immune reset plus

Antigen-specific immunotherapy (ASIT) has been used to control allergic diseases for over a century (24). However, despite clear evidence that this approach is effective in controlling experimental models of autoimmune diseases, it has been slow to translate to the clinic (25). The aim of ASIT is to ‘switch off’ pathogenic CD4^+^ T cells in a specific disease while simultaneously boosting self-antigen specific immunoregulatory T cells. This can be achieved through administration of self-antigens or their CD4^+^ T cell epitopes (26). A variety of administration routes and modes of delivery are in development; in essence, these different approaches all aim to target the self-antigens or self-epitopes to tolerance promoting cells, such as steady state dendritic cells (ssDC) in lymphoid organs or tolerance promoting immune environments such as the liver. Most importantly, the ASIT approaches being developed do not rely on B cells for their functional effect. For example, work from our laboratory has shown that antigenic epitopes designed to function as highly soluble, antigen processing independent peptides (PIPs) selectively bind ssDC in lymphoid organs following injection (27). PIPs preferentially bind to class II MHC molecules on ssDC since these cells do not load class II with peptide epitopes efficiently resulting in expression of unstable or peptide receptive class II MHC molecules at the cell surface (28). This means that peptide epitopes designed to bind MHC II in the appropriate conformation (PIPs) will bind to MHC II on ssDC rather than B cells or monocytes since the latter cells load MHC II efficiently and have stable MHC II at their cell surface. Critically, ssDC express low levels of costimulatory molecules (29); therefore, recognition of MHC II-peptide complexes on ssDC results in the induction of anergy in cognate, CD4^+^ helper cells and the propagation of antigen-specific regulatory T cells (27). Most importantly, tolerance induction with PIPs does not depend on B cells. Presentation of PIPs has been shown to promote differentiation of both Foxp3^+^ Treg cells and Tr1 cells in mice devoid of B cells (30). These antigen-specific regulatory T cell populations are capable of mediating bystander suppression whereby regulatory cells specific for antigen A of a tissue will suppress generation of cells specific for antigens B, C, D etc. from the same tissue (31).

While not yet proven formally, other delivery approaches for ASIT should also function in B cell depleted individuals based on their mode of action. Delivery of peptide antigens on aged red blood cells targets the antigens to the liver (32). Similarly, ferromagnetic nanoparticles designed for liver imaging have been shown to target liver sinusoidal endothelial cells (LSEC) (33). Presentation of self-antigens on LSEC promotes differentiation of Foxp3^+^ Treg cells in a TGF-β dependent fashion (34). Larger nanoparticles have been shown to target monocytes (35). These cells take up the antigen-loaded nanoparticle, migrate to the spleen and liver where they undergo apoptosis and release their antigen. T cell epitopes can be modified with sugar side chains to promote uptake in the liver without the need for nanoparticles for their delivery (36). Finally, peptide epitopes can be presented by artificial APCs prepared by coating nanoparticles with MHC class II molecules (37). These artificial APCs do not express costimulatory molecules: previously activated T cells encountering their peptide-MHC ligand in this form become anergic and differentiate into IL-10 secreting Tr1 cells capable of bystander suppression.

Some of the above approaches have been tested in early phase clinical trials (38–42). Results of these trials has shown that ASIT for autoimmune diseases is well tolerated with evidence of efficacy in a range of diseases. Most importantly, none of the approaches discussed above should be dependent on B cells for their efficacy and, therefore, should function in B cell depleted individuals.

## Discussion

We hereby propose that ASIT with any one of the delivery approaches mentioned above would be effective in people treated with B cell depleting therapies. The clearest evidence in favour of this comes from our own work on PIPs. PIPs would be effective as a means of maintaining immune homeostasis and preventing autoimmune relapses in patients treated with B cell depleting strategies since this form of immunotherapy has been shown to be effective in mice without B cells (30). We propose that ASIT should be applied with or shortly after B cell depleting immunotherapy. This would allow the patient’s immune system to recover from B cell depletion while maintaining control of their autoimmune condition through induction of antigen-specific immune regulation. Arguably, B cell depletion and ASIT could be given at the same time since B cell depletion does not interfere with tolerance induction by ASIT, as previously shown with PIPs, while treatment with PIPs and other ASIT approaches should not interfere with B cell depletion. The resulting maintenance of immune homeostasis would mean that the patient would only require a single cycle of B cell depletion after which immune tolerance would be maintained by regular administration of PIPs derived from relevant self-antigens. Our previous studies in experimental animals revealed that treatment with PIPs induced tolerance that lasted between 1 and 3 months in euthymic animals (43). Similar observations were made in clinical trials of PIP treatment for Graves’ disease (40) and relapsing MS (39). These studies demonstrated stable suppression of disease for up to a month after which some patients relapsed. We propose that safe and effective control of disease could be achieved by monthly administration of PIPs following B cell depletion and that this would achieve life-long immune reset. Most importantly, B cell populations would recover to homeostatic levels to provide protection from infection; furthermore, this strategy would allow effective vaccination in previously B cell depleted individuals. Autoreactive B cells would, however, remain starved of T cell help and would not expand or undergo class switching. This strategy would, therefore, control both cell and antibody mediated autoimmune diseases.
